# Altered spatiotemporal consistency in pediatric bipolar disorder patients with and without psychotic symptoms

**DOI:** 10.1186/s12888-021-03524-4

**Published:** 2021-10-15

**Authors:** Weijia Gao, Dong Cui, Qing Jiao, Linyan Su, Guangming Lu, Rongwang Yang

**Affiliations:** 1 Department of Child Psychology, The Children’s Hospital, Zhejiang University School of Medicine, National Clinical Research Center for Child Health, National Children’s Regional Medical Center, No. 3333 Binsheng Road, Zhejiang, Hangzhou China; 2 Institute of Biomedical Engineering, Chinese Academy of Medical Science & Peking Union Medical College, Tianjin, China; 3 Department of Radiology, Shandong First Medical University & Shandong Academy of Medical Sciences, Tai’an, Shandong China; 4 Mental Health Institute, The Second Xiangya Hospital of Central South University, Key Laboratory of Psychiatry and Mental Health of Hunan Province, National Technology Institute of Psychiatry, Changsha, Hunan China; 5 Department of Medical Imaging, Jinling Hospital, Clinical School of Medical College, Nanjing University, 305 Zhongshan East Road, Nanjing, Jiangsu China

**Keywords:** Pediatric bipolar disorder, Psychotic symptom, FOCA

## Abstract

**Objective:**

Psychotic symptoms are quite common in patients with pediatric bipolar disorder (PBD) and may affect the symptom severity and prognosis of PBD. However, the potential mechanisms are less well elucidated until now. Thus, the purpose of this study was to investigate the brain functional differences between PBD patients with and without psychotic symptoms.

**Method:**

A total of 71 individuals including: 27 psychotic PBD (P-PBD), 25 nonpsychotic PBD (NP-PBD), and 19 healthy controls were recruited in the present study. Each subject underwent 3.0 Tesla functional magnetic resonance imaging scan. Four-dimensional (spatiotemporal) Consistency of local neural Activities (FOCA) was employed to detect the local brain activity changes. Analyses of variance (ANOVA) were used to reveal brain regions with significant differences among three groups groups of individuals, and inter-group comparisons were assessed using post hoc tests.

**Results:**

The ANOVA obtained significant among-group FOCA differences in the left triangular inferior frontal gyrus, left supplementary motor area, left precentral gyrus, right postcentral gyrus, right superior occipital gyrus, and right superior frontal gyrus. Compared with the control group, the P-PBD group showed decreased FOCA in the left supplementary motor area and bilateral superior frontal gyrus and showed increased FOCA in the left triangular inferior frontal gyrus. In contrast, the NP-PBD group exhibited decreased FOCA in the right superior occipital gyrus and right postcentral gyrus and showed increased FOCA in the left orbital inferior frontal gyrus. Compared to the NP-PBD group, the P-PBD group showed decreased FOCA in the right superior frontal gyrus.

**Conclusion:**

The present findings demonstrated that the two groups of PBD patients exhibited segregated brain functional patterns, providing empirical evidence for the biological basis of different clinical outcomes between PBD patients with and without psychotic symptoms.

## Introduction

Pediatric bipolar disorder (PBD) receives more and more attention owing to the atypical symptoms, prolonged disease duration, more functional impairments, and resistance to treatment [1]. In a recent meta-analysis, the prevalence of PBD was estimated to be about 3.9% [2]. It was reported that approximately 50% of patients with bipolar disorder (BD) experienced psychotic symptoms, such as hallucinations, delusions, and other symptoms during their disease course [3], while the prevalence of psychotic symptoms in individuals with PBD was even higher [4]. The occurrence of psychotic symptoms could lead to significant impacts on clinical features and prognosis of BD [5]. Therefore, preliminary evidence has suggested that BD with psychotic symptoms can be treated as a specific phenotype with unique pathophysiology [6], although it was less well elucidated.

A growing body of studies are paying attention to the relationship between neuroimaging biomarkers and psychotic features in BD [7]. Regarding structural neuroimaging, results have suggested the presence of peculiar gray matter volume (GMV) differences between BD patients with and without psychotic symptoms. Psychotic BD seems to be associated with cortical GMV deficits compared to both healthy controls and non-psychotic BD patients, mainly in the frontal region. Conversely, non-psychotic BD patients showed GMV deficits in selective regions of the basal ganglia when compared with the other groups [8]. Our recent study focusing on PBD also found that PBD patients with psychotic features were associated with extensive structural abnormalities mainly located in the cortical-subcortical-limbic network, whereas non-psychotic PBD patients exhibited GM deficits in limited cortical regions [9]. In an attempt to clarify the brain functional alterations associated with the psychotic dimension of BD, a prior study using Global Brain Connectivity (GBC) method demonstrated that BD patients exhibited reduced medial prefrontal cortex (mPFC) connectivity, increased amygdala-mPFC connectivity, and reduced connectivity between amygdala and dorsolateral PFC. However, further analysis revealed that these effects were more pronounced in BD patients with psychotic symptoms. Moreover, the magnitude of observed effects was significantly correlated with lifetime psychotic symptom severity [10]. Another seed-based functional connectivity study revealed that ventral anterior cingulate cortex (vACC) connectivity alterations in BD patients depended on co-occurrence of lifetime psychosis [11]. Taken together, these findings highlight the importance of studies that focus on the effect of psychotic dimension on neuroimaging alterations in BD patients.

Resting state functional magnetic resonance imaging (fMRI) is a fabulous way to characterize the baseline brain activities without any tasks and can minimize the effect of external stimuli [12]. Currently, an increasing number of literatures have indicated that resting state functional alterations are most likely associated with the psychotic dimension in adults with BD; however, these associations were less well revealed in PBD patients. To the best of our knowledge, only one study has explored the influence of psychotic symptoms on default mode network (DMN) connectivity in PBD patients and found that psychotic PBD, but not PBD without psychotic symptoms, showed aberrant DMN connectivity relative to healthy controls [13]. In this context, the purpose of the present study was to investigate the brain resting state functional differences between PBD patients with and without psychotic symptoms. In the present study, a novel measure, named Four-dimensional (spatiotemporal) Consistency of local neural Activities (FOCA) [14], was applied to investigate the local spontaneous brain activity by integrating the temporal and spatial information of local brain regions. Temporal correlation can be used to reflect the temporal coherence of local neighboring voxels, while the spatial correlation was obtained to reflect the stability of regional activity in adjacent time points. The FOCA may reflects not only the consistency of the time course of adjacent voxel activities in the local brain region, but also the local brain region voxel activity at the adjacent time point is spatially consistent. A larger FOCA value means higher consistency of local spontaneous activity [15]. Based on previous findings, we hypothesized that psychotic symptoms could lead to different brain functional patterns mainly in the frontal regions in patients with PBD.

## Method

### Participants

Fifty-six PBD patients participated in the study, four cases were exclude from analysis because of poor scan quality. Then, a total of 52 children and adolescents, including 27 patients with psychotic PBD (P-PBD group) and 25 patients with non-psychotic PBD (NP-PBD group) were recruited from the child and adolescent psychiatric clinic of The Second Xiangya Hospital of Central South University, Changsha, Hunan, P.R. China from July, 2012 to August, 2013. For assignment to the P-PBD group, patients must have experienced a history (past or current) of psychotic symptoms during their illness period. Meanwhile, 19 age- and sex-matched healthy controls (HCs) were also recruited through advertisements in public schools. The inclusion criteria for PBD patients included: 1) aged 10–18 years, 2) met the Diagnostic and Statistical Manual of Mental Disorders, IV Edition (DSM- IV) criteria for BD, 3) the Han ethnicity, 4) right-handedness, 5) could follow the instructions to keep still during MRI scanning. Meanwhile, 19 age- and sex-matched healthy controls (HC) also were recruited through advertisements in public schools. The general exclusion criteria were as follows: 1) existence of major sensorimotor handicaps, 2) current active physical disease, 3) full-scale intelligence quotient (IQ) < 80, 4) contraindications to MRI scan, including retractors or braces, metallic implants, and claustrophobia, 5) presence of other mental disorders, such as schizophrenia, anorexia, bulimia nervosa, and learning disabilities, 6) drug or alcohol dependence or abuse, 7) with histories of electroconvulsive therapy (ECT). This study is one of our serial investigations focusing on PBD patients with and without psychotic symptoms, and the recruitment of participants has been described in our previous study [9].

### Clinical assessments

All subjects and at least one of their guardians were enrolled in a diagnostic interview using a clinical assessment combined with the Schedule for Affective Disorders and Schizophrenia for School-aged Children-Present and Lifetime Versions (K-SADS-PL). All the comorbidities were assessed by the K-SADS-PL interview according to the DSM- IV criteria as well. Each diagnosis was made by two experienced psychiatrists (Gao and Su) independently with satisfactory agreement (*kappa* = 0.85) for the inter-rater reliability. The P-PBD patients were defined as having experienced psychotic symptoms, such as delusions and/or hallucinations, in at least one prior episode. The NP-PBD patients were defined as the absence of psychotic symptoms in all episodes. The demographic and clinical data from all patients and controls were collected by a self-designed questionnaire. Current severity of mood symptoms were evaluated by Young Mania Rating Scale (YMRS) [16] and Mood and Feelings Questionnaire (MFQ) [17] on the day of MRI scanning, the clinical threshold points for the YMRS and MFQ were 12 and 18, respectively.

### MRI acquisition

The structural and resting-state functional data of all subjects were collected by Siemens 3.0-T MRI scanner (Allegra, Siemens Medical System). To ensure the quality of the data collected, the subjects were required to lie on the MRI scanner bed throughout the scan and to remain completely motionless, eyes closed and awake. To reduce the effects of noise, earplugs were worn on both ears during MRI data acquisition. The regular axial three-dimensional T1-weighted images were acquired with a spoiled gradient recall sequence with the following imaging parameters: repetition time (TR) = 2300 ms, echo time (TE) = 2.03 ms, gap = 0 mm, slice thickness = 1 mm, flip angle = 9°, matrix size = 256 * 256, field of view (FOV) = 256 * 256 mm^2^, slices = 176. An echo planar imaging sequence was applied to acquire the functional images, the parameters were as follows: TR = 2000 ms, TE = 30 ms, slice thickness = 4 mm, matrix = 64 × 64, FOV = 240*240 mm^2^, flip angle = 90°, gap = 0.4 mm, 30 axial slices, and a total of 250 volumes were collected for each participant.

### FOCA analysis

The data were preprocessed using the SPM8 software (http://www.fil.ion.ucl.ac.uk/spm/software/spm8/). During this process, the first 10 time points were removed to reduce the influence of the T1 saturation effects on the data quality. The Subjects whose head motion exceeded 1° or/and 1 mm were excluded. The realigned images were spatially normalized to the Montreal Neurological Institute (MNI) template and resliced with voxel size of 3 × 3 × 3 mm^3^. Following normalization, several spurious sources of variation—including head motion, linear drift, white matter (WM), and cerebrospinal fluid (CSF) signals were regressed out using multiple linear regression analysis. The FOCA then were calculated using the neuroscience information analysis tool NIT (http://www.neuro.uestc.edu.cn/NIT.html) [14]. The detailed calculation steps of FOCA were as follows: FOCA reflects the spatio-temporal consistency of local spontaneous activity in the brain. It is necessary to calculate the time correlation coefficient and the spatial correlation coefficient of each voxel. The FOCA value of each voxel was obtained by multiplying the temporal correlation coefficient by the spatial correlation coefficient. Then the average FOCA value of the whole brain was obtained on the basis of the FOCA value of each voxel. The normalized FOCA value was calculated by dividing the FOCA value of each voxel by the average FOCA value of the whole brain. The standardized FOCA value was subtracted by 1, and its image was spatially smoothed (FWHM: 6 mm).

### Statistical analysis

The statistical analyses of the demographic and clinical data were done using Statistical Package for the Social Sciences (SPSS) version 16.0 (SPSS Inc., Chicago, IL, USA). Chi-square tests (*χ*^*2*^) and analyses of variance (ANOVAs) were used to compare groups on non-parametric data and parametric data, respectively. The level of two-tailed statistical significance was set at *p* < 0.05 for all tests.

For FOCA comparisons, one-way ANOVA was calculated on the individual imaging maps in a voxel-by-voxel manner, and then a mask was built according to the results of ANOVA. Finally, the inter-group differences were assessed by using post hoc analyses based on the mask. The underlying confounders, such as age, educational level, and sex were included as covariates in the measures. The significance level was set at *p* < 0.05, AlphaSim corrected (combined height threshold *p* < 0.001 and cluster > 108) was analyzed with DPABI (http://rfmri.org/dpabi). Lastly, the average FOCA values of the clusters with group differences were extracted by using region of interest (ROI) analyses. The correlations of FOCA alterations with clinical characteristics, including illness course, onset age, episode times, and scores of YMRS and MFQ, were assessed by performing Pearson’s correlation analyses.

## Result

### Demographic and clinical features

The demographic and clinical characteristics of subjects in the three groups are listed in Table 1. The three groups of individuals did not differ with respect to age, sex, years of education, and IQ. Nevertheless, the three groups differed on the average scores of YMRS and MFQ. Compared to HCs, patients in P-PBD group showed higher scores on YMRS and MFQ, whereas patients with NP-PBD scored higher on YMRS only. There was no significant difference between two groups of patients on distributions of PBD type, current mood state, comorbidity, family history, means of illness duration, onset age, and episode times. A total of 15 (55.6%) P-PBD patients reported current psychotic symptoms, while the rest (12, 44.4%) in P-PBD group reported only past history of psychotic symptoms. A higher proportion of P-PBD patients (88.9%) received drugs for treatment compared to the NP-PBD group (56.0%) at the time of recruitment.

**Table 1:** Demographic and clinical characteristics of all subjects (*n* = 71)

	P-PBD group *n* = 27	NP-PBD group *n* = 25	HC group *n* = 19	*F/χ*^*2*^	*p*
Age (Years)	15.4 (1.55)	14.8 (1.94)	14.2 (1.57)	2.858	0.064
Sex (Male/Female)	11/16	14/11	7/12	1.930	0.381
Education (Years)	8.37 (1.50)	7.96 (2.11)	7.47 (2.22)	1.204	0.306
IQ	102.9 (13.8)	105.0 (12.5)	105.3 (7.51)	0.311	0.733
Illness course (Months)	16.7 (12.3)	17.3 (16.8)		0.018	0.895
Onset age (Years)	14.2 (1.54)	13.3 (2.09)		2.939	0.093
Episode times	3.59 (1.65)	4.71 (7.09)		0.631	0.431
PBD-I/PBD-II	16/11	17/8		0.428	0.513
YMRS	12.4 (13.1)^*b*^	15.4 (14.4)^*a*^	3.63 (2.06)	5.437	0.006
MFQ	15.2 (14.2)^*a*^	11.7 (10.6)	6.11 (3.33)	3.862	0.026
Psychotic symptom, n (%)					
Current	15 (55.6)				
Past	12 (44.4)				
Current status, n (%)					
Mania	8 (29.6)	9 (36.0)		0.239	0.625
Depression	8 (29.6)	7 (28.0)		0.017	0.897
Euthymia	11 (40.8)	9 (36.0)		0.318	0.573
Family history (Y/N)	5/22	7/18		0.657	0.417
Treatment, n (%)					
None	3 (11.1)	11 (44.0)		7.137	0.008
Lithium	12 (44.4)	7 (28.0)		1.514	0.219
Valproate	15 (55.6)	9 (36.0)		1.997	0.158
Antipsychotics	22 (81.5)	12 (48.0)		11.15	0.001
Antidepressants	2 (7.41)	1 (4.0)		0.272^*^	0.602
Comorbidity n (%)					
Anxiety	7 (25.9)	3 (12.0)		1.590^*^	0.207
ADHD	2 (7.41)	1 (4.0)		0.272^*^	0.602
OCD	1 (3.70)	1 (4.0)		0.003^*^	0.956

*ADHD* attention deficit hyperactivity disorder, *HC* healthy controls, *IQ* intelligence quotient, *MFQ* mood and feelings questionnaire, *NP-PBD* nonpsychotic pediatric bipolar disorder, *OCD* obsessive-compulsive disorder, *P-PBD* psychotic pediatric bipolar disorder, *YMRS* Young Mania Rating Scale. ^*a*^ Compared with HC, *p* < 0.01, ^*b*^ Compared with HC *p* < 0.05, ^*^Fisher’s exact test

### Alterations of FOCA

**Fig. 1:**
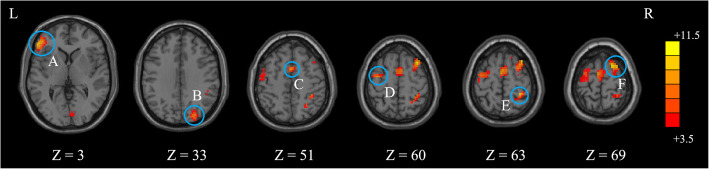
Brain regions showing significant functional differences among three groups (*p* < 0.05, *AlphaSim corrected*). The color scale represents *F* values of ANOVA. (**A**) the left Triangular IFG; (**B**) the right SOG; (**C**) the left SMA; (**D**) the left Precentral gyrus; (**E**) the right Postcentral gyrus; (**F**) the right SFG

**Fig. 2:**
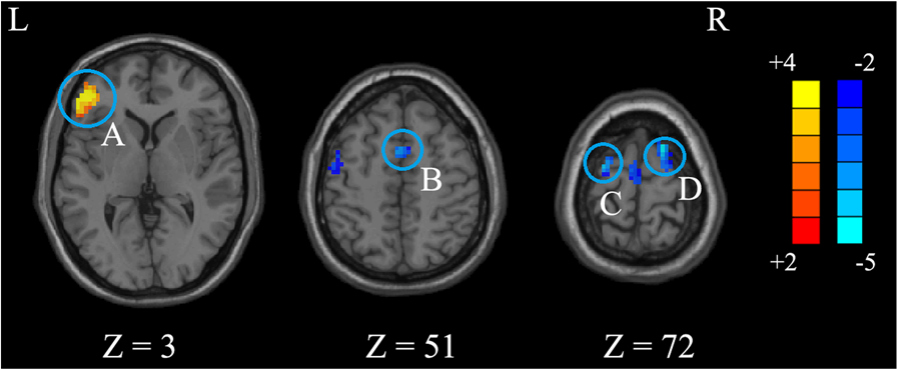
Brain functional differences between P-PBD and HCs (*p* < 0.05, *AlphaSim corrected*). The color scale represents *t* values. (**A**) the left Triangular IFG; (**B**) the left SMA; (**C**) the left SFG; (**D**) the right SFG

**Fig. 3:**
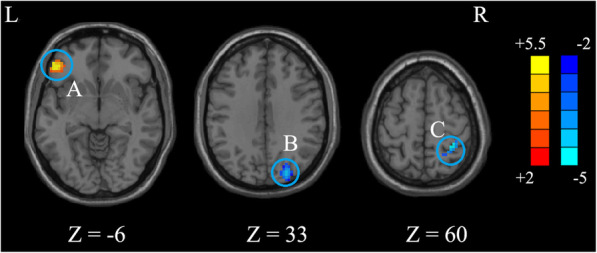
Brain functional differences between NP-PBD and HCs (*p* < 0.05, *AlphaSim corrected*). The color scale represents *t* values. (**A**) the left Orbital IFG; (**B**) the right SOG; (**C**) the right Postcentral gyrus

**Fig. 4:**
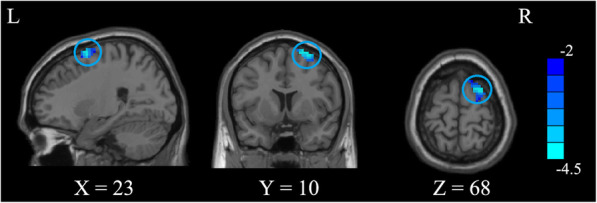
Brain functional differences between P-PBD and NP-PBD (*p* < 0.05, *AlphaSim corrected*). The color scale represents *t* values

The ANOVA indicated that significant FOCA differences were found among three groups. These differences were located in the left triangular inferior frontal gyrus (IFG), left supplementary motor area (SMA), left precentral gyrus, right postcentral gyrus, right superior occipital gyrus (SOG), and right superior frontal gyrus (SFG) (Table 2 and Fig. 1). Compared to the control group, the P-PBD group showed decreased FOCA in the left SMA and bilateral SFG and showed increased FOCA in the left triangular IFG (Table 3 and Fig. 2). In contrast, the NP-PBD group exhibited decreased FOCA in the right SOG and right postcentral gyrus and showed increased FOCA in left orbital IFG (Table 3 and Fig. 3). Compared to the NP-PBD group, the P-PBD group showed decreased FOCA in the right SFG (Table 3 and Fig. 4). The mean cluster values of the regions that showed significant differences in the above FOCA analysis were extracted from each patient. However, no significant association was found between FOCA alterations and clinical features in PBD patients.

**Table 2:** Brain regions showing significant functional differences among three groups (*p* < 0.05, *AlphaSim corrected*)

Brain region	Hemisphere	Cluster size	***F*** value	MNI coordinate		
				***x***	***y***	***z***
Triangular IFG	L	199	8.72	−54	36	3
Postcentral gyrus	R	225	8.93	30	−39	63
SOG	R	118	7.17	24	−84	33
SMA	L	168	8.25	0	6	51
Precentral gyrus	L	161	7.50	−36	−6	60
SFG	R	165	11.4	18	9	69

*IFG* Inferior frontal gyrus, *MNI* Montreal neurological institute, *SFG* Superior frontal gyrus, *SMA* Supplementary motor area, *SOG* Superior occipital gyrus

**Table 3:** Brain regions showing inter-group differences based on the results of ANOVA (*p* < 0.05, *AlphaSim corrected*)

Brain region	Hemisphere	Cluster size	***t*** value	MNI coordinate		
				***x***	***y***	***z***
**P-PBD < HC**						
SMA	L	166	−3.83	0	6	51
SFG	L	113	−4.14	−27	−3	72
	R	121	−4.77	18	9	72
**P-PBD > HC**						
Triangular IFG	L	188	3.73	− 45	42	3
**NP-PBD < HC**						
SOG	R	111	−4.15	24	−84	33
Postcentral gyrus	R	137	−4.71	33	−39	60
**NP-PBD > HC**						
Orbital IFG	L	138	5.21	−51	39	−6
**P-PBD < NP-PBD**						
SFG	R	159	−4.24	23	10	68

*ANOVA* analyses of variance, *HC* health controls, *IFG* Inferior frontal gyrus, *MNI* Montreal neurological institute, *NP-PBD* nonpsychotic pediatric bipolar disorder, *P-PBD* psychotic pediatric bipolar disorder, *SFG* Superior frontal gyrus, *SMA* Supplementary motor area, *SOG* Superior occipital gyrus

## Discussion

In this study the FOCA method was used to distinguish the resting-state brain function between PBD patients with and without psychotic symptoms. Our findings demonstrated that the two PBD groups exhibited similar, but mostly distinct brain functional abnormalities. The differences in brain functional alterations are likely to be part of the underlying neurobiological basis for the presence of psychotic symptoms. Thus, these findings add to the literature on novel pathogenesis involvement in PBD patients with and without psychotic symptoms and support the viewpoint that individual differences in history of psychotic symptoms should seriously be considered in future studies of PBD.

Brain functional changes in the prefrontal cortex (PFC) have frequently been reported in previous resting-state or task-related fMRI studies in PBD. In a meta-analysis of fMRI studies, when engaged in attentional tasks, increased activation was observed in the IFG with decreased activation in the limbic regions in patients with PBD, relative to typically developing subjects [18]. In addition, our prior studies also demonstrated that PBD patients exhibited functional disturbances in the prefrontal regions, including the IFG, SFG, orbital frontal gyrus (OFG), medial frontal gyrus, and middle frontal gyrus (MFG) [12, 19, 20]. The present study found that PBD patients with psychotic symptoms showed more extensive functional changes in the prefrontal cortex (PFC), especially in the bilateral SFG and left triangular IFG, whereas the NP-PBD group exhibited FOCA changes only in the left orbital IFG. The role of the PFC in psychosis has been suggested by a recent systematic review of adult BD neuroimaging studies. While not entirely consistent, comparisons between P-BD and NP-BD patients suggested that gray matter volume alterations mainly involved the PFC, such as the left SFG, right MFG, and left IFG [21]. Taken together, our current findings provided new implications that psychotic features might have effects on the PFC functioning in PBD patients as well.

In our study, when comparing to HCs, both groups of PBD patients showed increased FOCA in the left IFG. It has been demonstrated that dysfunction of the IFG is implicated in emotional dysregulation and executive disturbance [22], which are core characteristics of BD. Prior studies have reported that abnormal functioning of the IFG during cognitive or emotion-inducing tasks are commonly observed in both adult BD and PBD patients [18, 23, 24]. Furthermore, the IFG also works in concert with different brain regions both in terms of cognitive and emotional regulation functions, highlighting the importance of network abnormalities in BD. In a recent study, patients with PBD were revealed to exhibit increased functional connectivity between IFG and ventromedial prefrontal cortex (vmPFC) following feedback processing, as well as elevated functional connectivity between IFG and parahippocampal gyrus (PHG)/periaqueductal gray (PAG) during attention orienting following frustration [25]. In general, we speculated that the current finding of regional activation of the IFG might be associated with mood dysregulation and executive deficits in patients with PBD.

A second noteworthy finding was that group difference of resting-state brain function between PBD patients with and without psychotic symptoms was observed in the right SFG. Current PBD diagnosis combined with psychotic symptoms history seemed to be good predictors of dysfunction in the right SFG. The SFG is a part of the dorsolateral prefrontal cortex (DLPFC). The DLPFC plays a key role in plenty of mental processing, including executive functioning, attention, emotional processing, and working memory [26]. Functional disruptions in the DLPFC have been proved to account for working memory deficits and emotional dysregulations in BD patients with psychotic symptoms and schizophrenia [27, 28]. Our finding was, at least in part, comparable with a prior study reporting that functional connectivity of the frontoparietal control network, in which the DLPFC was a key component, was disrupted in multiple psychotic disorders, including schizophrenia and psychotic BD [29]. Interestingly, the investigators further found that the presence of effective and psychotic symptoms might lead to graded disruptions in frontoparietal network connectivity in their latter work. Moreover, there was a link between psychotic symptoms and decreased frontoparietal network connectivity [30]. Based on these findings, molecular mechanisms involving the DLPFC in psychotic BD also were revealed. A recent postmortem study exploring the proteomic differences associated with BD psychosis in the DLPFC indicated that psychotic symptoms in patients with BD might be associated with abnormalities in neurodevelopment, neuroplasticity, neurotransmission, and neuromodulation in the DLPFC [31]. Taken together, the extant evidence may suggest that the DLPFC could be a potential region for distinguishing BD patients with and without psychotic symptoms.

In the present study FOCA alterations were found also in the primary sensorimotor regions, such as the left SMA and right postcentral gyrus in PBD patients. The SMA has a wide range of connections to many brain areas, such as the IFG, thalamus, and striatum. These related brain areas play a key role in motor control, action selection, preparation, motor execution, and internally generated movements [32]. A previous study reported that children with BD exhibited decreased SMA volume when comparing to healthy controls [33]. Increased regional homogeneity (ReHo) in the right SMA was found in unmedicated patients with BD II depression as well [34]. In addition, abnormal functional connectivity between the DMN and the motor area network was reported in the euthymic BD patients. The authors suggested that these brain function alterations might be the potential neurobiological underpinnings for their patients’ residual manic symptoms, such as psychomotor agitation or psychotic symptomatology [35]. In our study only PBD patients with psychotic symptoms showed decreased FOCA in the left SMA when compared to healthy controls; thus, we speculated that functional changes of the SMA might be more likely to represent the biological mechanisms underlying psychotic and psychomotor symptoms in PBD.

Some limitations should be noted in the present study. First, for the participants, the sample size of each subgroup was relatively small and restricted us to discuss the effects of psychotic symptoms on brain functional changes in a single type (PBD I, or PBD II) or mood state (mania, depression, or euthymia), it should be taken more seriously, because in a series of our past studies focusing on PBD, we have already observed different patterns of brain function during different mood state [36, 37]. Meanwhile, the age range 10–18 might be pretty extensive, which could lead to brain functional differences independent of the diagnosis [38]. Second, the majority of the PBD patients were taking medications when scanning, but the effects of medication use on brain functional changes remained unknown. Considering the small sample of the present study, we did not study this issue deeply, however, a previous review revealed that medication appeared to influence many structural MRI studies, but had limited impact on functional MRI and Diffusion Tensor Imaging (DTI) findings [39]. Third, there were still many confounders that may have an influence on brain function (e.g., comorbidity and family history), and they should be well controlled in feature studies. Forth, cognitive function was not assessed in the present study, since cognitive deficits could be an additional way of differentiating the two groups of PBD patients [40] and it might also be associated with brain function alterations, cognitive tests should be added in feature studies as well. Finally, the cross-sectional design of this study precluded any causal inference.

## Conclusion

In summary, the present findings demonstrated that the two groups of PBD patients exhibited segregated brain functional patterns, providing empirical evidence for the biological basis of different clinical outcomes between PBD patients with and without psychotic symptoms. Individual differences in histories of psychotic symptoms should seriously be considered in future neuroimaging studies of PBD.

## Data Availability

The data will be available from the corresponding author on reasonable request.

## References

[1] Liberg B, Ekman CJ, Sellgren C, Johansson AG, Landen M (2015). Subcortical morphometry and psychomotor function in euthymic bipolar disorder with a history of psychosis. Brain Imag Behav.

[2] Van Meter A, Moreira ALR, Youngstrom E: Updated Meta-Analysis of Epidemiologic Studies of Pediatric Bipolar Disorder. The Journal of clinical psychiatry 2019;80(3):18r12180. 10.4088/JCP.18r12180. 10.4088/JCP.18r12180 30946542

[3] Dunayevich E, Keck PE (2000). Prevalence and description of psychotic features in bipolar mania. Curr Psychiatr Rep.

[4] Pavuluri MN, Herbener ES, Sweeney JA (2004). Psychotic symptoms in pediatric bipolar disorder. J Affect Disord.

[5] Keck PE, McElroy SL, Havens JR, Altshuler LL, Nolen WA, Frye MA, Suppes T, Denicoff KD, Kupka R, Leverich GS (2003). Psychosis in bipolar disorder: phenomenology and impact on morbidity and course of illness. Compr Psychiatry.

[6] Buoli M, Caldiroli A, Cumerlato Melter C, Serati M, de Nijs J, Altamura AC (2016). Biological aspects and candidate biomarkers for psychotic bipolar disorder: a systematic review. Psychiatry Clin Neurosci.

[7] Ekman CJ, Petrovic P, Johansson AG, Sellgren C, Ingvar M, Landen M (2017). A history of psychosis in bipolar disorder is associated with gray matter volume reduction. Schizophr Bull.

[8] Maggioni E, Altamura AC, Brambilla P (2017). Exploring the neuroanatomical bases of psychotic features in bipolar disorder. Epidemiology and psychiatric sciences.

[9] Gao W, Cui D, Jiao Q, Su L, Yang R, Lu G (2021). Brain structural alterations in pediatric bipolar disorder patients with and without psychotic symptoms. J Affect Disord.

[10] Anticevic A, Brumbaugh MS, Winkler AM, Lombardo LE, Barrett J, Corlett PR, Kober H, Gruber J, Repovs G, Cole MW, Krystal JH, Pearlson GD, Glahn DC (2013). Global prefrontal and fronto-amygdala dysconnectivity in bipolar I disorder with psychosis history. Biol Psychiatry.

[11] Anticevic A, Savic A, Repovs G, Yang G, McKay DR, Sprooten E, Knowles EE, Krystal JH, Pearlson GD, Glahn DC (2015). Ventral anterior cingulate connectivity distinguished nonpsychotic bipolar illness from psychotic bipolar disorder and schizophrenia. Schizophr Bull.

[12] Gao W, Jiao Q, Lu S, Zhong Y, Qi R, Lu D, Xiao Q, Yang F, Lu G, Su L (2014). Alterations of regional homogeneity in pediatric bipolar depression: a resting-state fMRI study. BMC psychiatry.

[13] Zhong Y, Wang C, Gao W, Xiao Q, Lu D, Jiao Q, Su L, Lu G (2019). Aberrant resting-state functional connectivity in the default mode network in pediatric bipolar disorder patients with and without psychotic symptoms. Neurosci Bull.

[14] Dong L, Luo C, Cao W, Zhang R, Gong J, Gong D, Yao D (2015). Spatiotemporal consistency of local neural activities: a new imaging measure for functional MRI data. Journal of magnetic resonance imaging : JMRI.

[15] Dong L, Li H, He Z, Jiang S, Klugah-Brown B, Chen L, Wang P, Tan S, Luo C, Yao D (2016). Altered local spontaneous activity in frontal lobe epilepsy: a resting-state functional magnetic resonance imaging study. Brain and behavior.

[16] Young RC, Biggs JT, Ziegler VE, Meyer DA (1978). A rating scale for mania: reliability, validity and sensitivity. Br J Psychiatr.

[17] Wood A, Kroll L, Moore A, Harrington R (1995). Properties of the mood and feelings questionnaire in adolescent psychiatric outpatients: a research note. Journal of child psychology and psychiatry, and allied disciplines.

[18] Lee MS, Anumagalla P, Talluri P, Pavuluri MN (2019). Attentional engagement increases inferior frontal gyrus activity and mutes limbic activity in pediatric bipolar disorder: Meta-analyses of fMRI studies. Prog Neuro-Psychopharmacol Biol Psychiatry.

[19] Guo Y, Wang J, Jiao Q, Cao W, Cui D, Gao W, Qiu J, Su L, Lu G (2021). Altered spatiotemporal consistency of corticolimbic circuitry in euthymic pediatric bipolar disorder. Brain imaging and behavior.

[20] Xiao Q, Zhong Y, Lu D, Gao W, Jiao Q, Lu G, Su L (2013). Altered regional homogeneity in pediatric bipolar disorder during manic state: a resting-state fMRI study. PLoS One.

[21] Wang X, Tian F, Wang S, Cheng B, Qiu L, He M, Wang H, Duan M, Dai J, Jia Z (2018). Gray matter bases of psychotic features in adult bipolar disorder: a systematic review and voxel-based meta-analysis of neuroimaging studies. Hum Brain Mapp.

[22] Corbetta M, Shulman GL (2002). Control of goal-directed and stimulus-driven attention in the brain. Nat Rev Neurosci.

[23] Brotman MA, Tseng WL, Olsavsky AK, Fromm SJ, Muhrer EJ, Rutenberg JG, Deveney CM, Adleman NE, Zarate CA, Pine DS (2014). Fronto-limbic-striatal dysfunction in pediatric and adult patients with bipolar disorder: impact of face emotion and attentional demands. Psychol Med.

[24] Wegbreit E, Cushman GK, Puzia ME, Weissman AB, Kim KL, Laird AR, Dickstein DP (2014). Developmental meta-analyses of the functional neural correlates of bipolar disorder. JAMA psychiatry.

[25] Ross AJ, Roule AL, Deveney CM, Towbin KE, Brotman MA, Leibenluft E, Tseng WL (2021). A preliminary study on functional activation and connectivity during frustration in youths with bipolar disorder. Bipolar Disord.

[26] Schmahl CG, Vermetten E, Elzinga BM, Bremner JD (2004). A positron emission tomography study of memories of childhood abuse in borderline personality disorder. Biol Psychiatry.

[27] Jenkins LM, Bodapati AS, Sharma RP, Rosen C (2018). Working memory predicts presence of auditory verbal hallucinations in schizophrenia and bipolar disorder with psychosis. J Clin Exp Neuropsychol.

[28] Jimenez-Lopez E, Sanchez-Morla EM, Lopez-Villarreal A, Aparicio AI, Martinez-Vizcaino V, Vieta E, Rodriguez-Jimenez R, Santos JL (2019). Neurocognition and functional outcome in patients with psychotic, non-psychotic bipolar I disorder, and schizophrenia. A five-year follow-up. Eur Psychiatr.

[29] Baker JT, Holmes AJ, Masters GA, Yeo BT, Krienen F, Buckner RL, Ongur D (2014). Disruption of cortical association networks in schizophrenia and psychotic bipolar disorder. JAMA Psychiatr.

[30] Baker JT, Dillon DG, Patrick LM, Roffman JL, Brady RO, Pizzagalli DA, Ongur D, Holmes AJ (2019). Functional connectomics of affective and psychotic pathology. Proc Natl Acad Sci U S A.

[31] Ho AM, Cabello-Arreola A, Markota M, Heppelmann CJ, Charlesworth MC, Ozerdem A, Mahajan G, Rajkowska G, Stockmeier CA, Frye MA (2020). Label-free proteomics differences in the dorsolateral prefrontal cortex between bipolar disorder patients with and without psychosis. J Affect Disord.

[32] Nachev P, Kennard C, Husain M (2008). Functional role of the supplementary and pre-supplementary motor areas. Nat Rev Neurosci.

[33] Adleman NE, Fromm SJ, Razdan V, Kayser R, Dickstein DP, Brotman MA, Pine DS, Leibenluft E (2012). Cross-sectional and longitudinal abnormalities in brain structure in children with severe mood dysregulation or bipolar disorder. Journal of child psychology and psychiatry, and allied disciplines.

[34] Qiu S, Chen F, Chen G, Jia Y, Gong J, Luo X, Zhong S, Zhao L, Lai S, Qi Z, Huang L, Wang Y (2019). Abnormal resting-state regional homogeneity in unmedicated bipolar II disorder. J Affect Disord.

[35] Bellani M, Bontempi P, Zovetti N, Gloria Rossetti M, Perlini C, Dusi N, Squarcina L, Marinelli V, Zoccatelli G, Alessandrini F, Francesca Maria Ciceri E, Sbarbati A, Brambilla P (2020). Resting state networks activity in euthymic bipolar disorder. Bipolar Disord.

[36] Xiao Q, Cui D, Jiao Q, Zhong Y, Cao W, Lu G, Su L (2019). Altered regional homogeneity in pediatric bipolar disorder during manic and euthymic state: a resting-state fMRI study. Brain imaging and behavior.

[37] Xiao Q, Wu Z, Hui X, Jiao Q, Zhong Y, Su L, Lu G (2021). Manic and euthymic states in pediatric bipolar disorder patients during an emotional go/Nogo task: a functional magnetic resonance imaging study. J Affect Disord.

[38] Damoiseaux JS (2017). Effects of aging on functional and structural brain connectivity. NeuroImage.

[39] Hafeman DM, Chang KD, Garrett AS, Sanders EM, Phillips ML (2012). Effects of medication on neuroimaging findings in bipolar disorder: an updated review. Bipolar Disord.

[40] Bora E (2018). Neurocognitive features in clinical subgroups of bipolar disorder: a meta-analysis. J Affect Disord.

